# Tuning the B-CLL microenvironment: evidence for BAG3 protein- mediated regulation of stromal fibroblasts activity

**DOI:** 10.1038/s41420-024-02153-6

**Published:** 2024-08-28

**Authors:** Anna Basile, Valentina Giudice, Laura Mettivier, Antonia Falco, Anna Lisa Cammarota, Angela D’Ardia, Carmine Selleri, Margot De Marco, Nicola De Maio, Maria Caterina Turco, Liberato Marzullo, Alessandra Rosati

**Affiliations:** 1 Department of Sanitary Hygiene and Evaluative Medicine U.O.C. Clinical and Microbiological Pathology, University Hospital “San Giovanni di Dio e Ruggi d’Aragona”, Salerno, Italy; 2 Department of Medicine, Surgery and Dentistry “Schola Medica Salernitana”, University of Salerno, Baronissi, Italy; 3 Hematology and Transplant Center, University Hospital “San Giovanni di Dio e Ruggi d’Aragona”, Salerno, Italy; 4 FIBROSYS s.r.l. Academic Spin-off, University of Salerno, Baronissi, Italy

**Keywords:** Cancer microenvironment, Chronic lymphocytic leukaemia

## Abstract

The Bcl2-associated athanogene-3 (BAG3) protein, a critical regulator of cellular survival, has been identified as a potential therapeutic target in various malignancies. This study investigates the role of BAG3 within stromal fibroblasts and its interaction with B-cell chronic lymphocytic leukemia (B-CLL) cells. Previous research demonstrated that BAG3 maintains the active state of pancreatic stellate cells (PSCs) and aids pancreatic ductal adenocarcinoma (PDAC) spread via cytokine release. To explore BAG3’s role in bone marrow-derived stromal fibroblasts, BAG3 was silenced in HS-5 cells using siRNA. In co-culture experiments with PBMCs from B-CLL patients, BAG3 silencing in HS-5 cells increased apoptosis and decreased phosphorylation of BTK, AKT, and ERK in B-CLL cells, thus disrupting their pro-survival key signaling pathways. The observation of fibroblast-activated protein (FAP) positive cells in infiltrated bone marrow specimens co-expressing BAG3 further support the involvement of the protein in fibroblast-mediated tumor survival. Additionally, BAG3 appears to support B-CLL survival by modulating cytokine networks, including IL-10 and CXCL12, which are essential for leukemic cell survival and proliferation. A robust correlation between BAG3 expression and the levels of CXCL12 and IL-10 was observed in both co-cultures and patient specimens. These findings point out the need for a more in-depth comprehension of the intricate network of interactions within the tumor microenvironment and provide valuable insights for the selection of new potential therapeutic targets in the medical treatment of CLL.

## Introduction

Chronic lymphocytic leukemia (CLL) is a mature B-cell tumor defined by the gradual accumulation of monoclonal B-cell lymphocytes. CLL accounts for 25–30% of all leukemia cases in Western countries, and the disease’s incidence increases exponentially with age, peaking in elderly populations [1]. Treatment options include watchful waiting [2], a combination of the B-cell lymphoma 2 (BCL2) inhibitor venetoclax with obinutuzumab, monotherapy with inhibitors of Bruton tyrosine kinase (BTK) such as ibrutinib and acalabrutinib, or chemoimmunotherapy [3]. For younger, fit patients with aggressive or relapsed CLL, stem cell transplantation may be an option [4]. The efficacy of these treatments varies depending on the disease stage, genetic alterations, and patient response. Some patients achieve long-term remission or cure, whilst others may relapse or develop resistance to therapy [3]. Although most therapeutic strategies employed in CLL hitherto have focused on targeting the leukemic cells, emerging evidence implies that modulation of microenvironmental cells and CLL–Tumor MicroEnvironment (TME) interactions by novel therapeutic agents could significantly affect their clinical efficacy [5, 6].

Indeed, the stromal microenvironment plays an important role in the pathogenesis of B-CLL, coordinating a complex signaling network that tightly governs malignant B cell survival, proliferation, and apoptosis evasion. The stromal constituents, which are primarily found in lymph nodes, the spleen, and bone marrow, are made up of fibroblasts as well as a variety of other supportive cellular elements such as nurse-like cells, endothelial cells, and T cells, all of which work together to create a nurturing environment that protects B-CLL cells from therapeutic interventions and immune surveillance mechanisms [7]. In a dynamic exchange, B-CLL cells communicate bidirectionally with stromal counterparts, actively affecting the TME to their advantage. B-CLL cells initiate stromal remodeling by direct cell-to-cell contacts and the production of a variety of autocrine and/or paracrine signaling molecules, establishing an environment permissive to disease progression. This reciprocal interaction not only preserves the leukemic niche, but also ensures the disease’s chronic development [8].

Bone marrow stromal cells have the remarkable ability to transform into the cancer-associated fibroblast (CAF) phenotype, making them an important component of the TME and exerting significant influence through the secretion of cytokines that promote tumor cell proliferation [9]. CAF activation is characterized by increased expression of alpha-smooth muscle actin (α-SMA), which correlates with increased proliferative and migratory capabilities. This has a significant impact on cancer cells, especially those with hematologic malignancies [10].

The quest for novel insights into the molecular determinants governing fibroblast activation is critical in the context of B-CLL, as it holds the promise of revealing novel diagnostic biomarkers and therapeutic targets that have the potential to transform patient prognosis and treatment outcomes.

BAG3 (Bcl2-associated athanogene-3), a multifunctional protein found in many tissues, is particularly abundant in cardiac and skeletal muscle tissues, as well as a variety of cancers. Within the complex environment of cancer biology, BAG3 emerges as a key player, regulating a wide range of cellular events required for tumor growth and survival [11, 12]. Indeed, growing data in oncology highlights the critical role of BAG3 in promoting cancer cell proliferation and resilience. Furthermore, in the context of pancreatic ductal adenocarcinoma (PDAC), BAG3 is also expressed in activated pancreatic stellate cells (PSCs), where it actively contributes to the maintenance of their activated state and facilitates PDAC invasion by orchestrating the release of a variety of cytokines [13]. Notably, BAG3’s influence extends beyond intracellular context, as it is released by pancreatic cancer cells, causing a paracrine effect on neighboring fibroblasts. Through the binding to its IFITM2 receptor [14], BAG3 activates fibroblasts, and induces the expression of α-SMA [15], cytokine secretion [16], and extracellular matrix deposition within the TME [17]. This complicated interplay emphasizes the multifaceted nature of BAG3’s role in tumor formation and its potential as a therapeutic target in the ongoing battle against cancer.

This study contributes further insights in the effort to clarify the role of BAG3 in stromal fibroblasts and in the complex network of interactions with B- chronic lymphocytic leukemia (B-CLL) cells, to better understand its envisionable critical role in the regulation of soluble mediators required for leukemic cancer cell viability.

## Results

### BAG3 expression supports stromal fibroblasts-induced survival in primary B-CLL cells

**Fig. 1:**
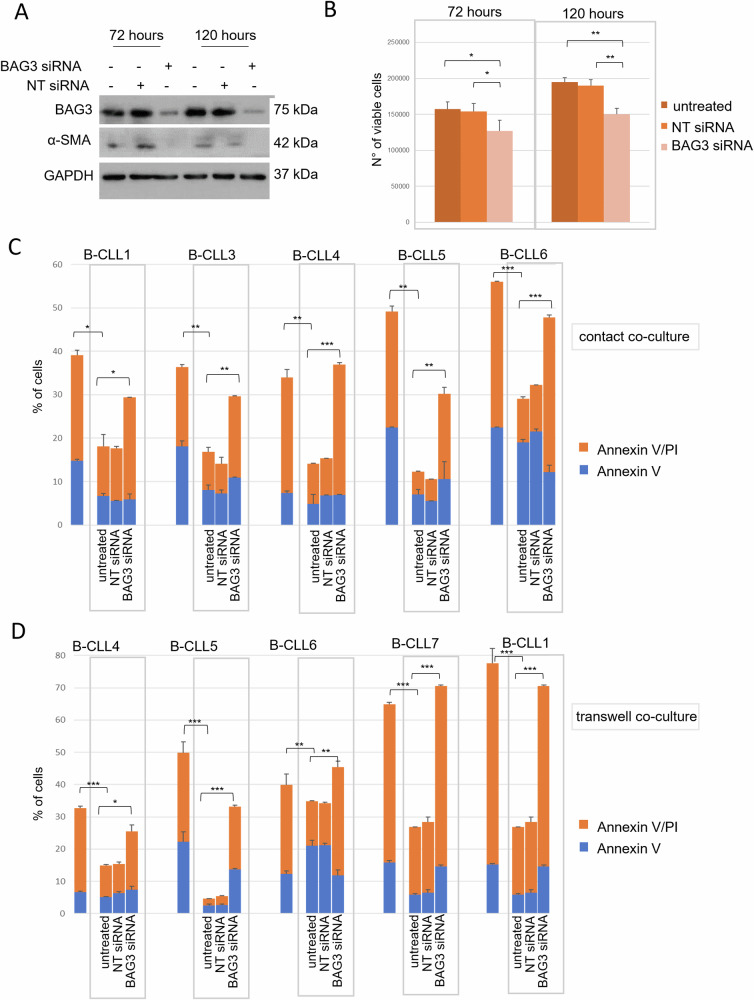
BAG3 silencing negatively affects tumor survival supported by stromal fibroblasts. **A** HS-5 cells (80% confluence) were transfected with either BAG3 siRNA or non-target (NT) siRNA. After 72 h and 120 h, total protein extracts were immunoblotted with anti-BAG3 polyclonal antibody, anti-α-SMA monoclonal antibody, or control anti-GAPDH antibody. **B** The number of viable cells after transfection was determined using trypan blue exclusion. **C**, **D** HS-5 cells were transfected with either BAG3 siRNA or NT siRNA (NT). After 16 h, patient-derived B-CLL cells were exposed to transfected HS-5 in both contact (**C**) and transwell (**D**) co-culture conditions. After 120 h, the B-CLL was collected and incubated with APC-anti-CD19 for 15 min before being double-stained with annexin V-FITC and PI under non-permeabilized conditions and analyzed using flow cytometry. Data are presented as mean ± S.D. of duplicate experiments. Student’s *t*-test was used to calculate *p* values, which are represented as *p < 0.05 > 0.01, **p < 0.01 > 0.001, and ***p < 0.001.

A previous study showed that BAG3 maintains the active state of PSCs and stimulates PDAC cells to spread by releasing different cytokines [13]. In fact, IL-8, MCP1, TGF-β2, and IGFBP2 are released by BAG3-positive PSCs and can promote PDAC invasion through their paracrine stimulation. To study the role of BAG3 in bone marrow-derived stromal fibroblast activation, the expression of the protein was silenced by siRNA experiments in HS-5 cells. This cell line has been recognized as an appropriate and widely accepted fibroblast model for in vitro studies of stroma-B-CLL molecular interactions and for its ability to mimic tumor biology in vivo, particularly for the stromal influence in the affected tissues [18–21]. According to previously published data obtained in other fibroblast cell lines, the down-modulation of BAG3 in HS-5 cells for 72 h and 120 h resulted in a parallel progressive decrease of alpha-SMA expression (Fig. 1A), a marker of fibroblast activation. Furthermore, BAG3 silencing also produced a slight, but significant, reduction of cell viability at both 72 h (−19.7% ± 5.8; mean% ± S.D.) and 120 h (−23.0% ± 4.9), in comparison to untreated cells or cells transfected with a non-targeting-(NT) siRNA. The percentage of dead cells was minimal and uniform in all the experimental settings and checked time points (data not shown).

Stromal fibroblasts secrete pro-inflammatory cytokines and growth factors, which can significantly affect B-CLL cell biology. The effect of BAG3 silencing was then studied in co-cultures of HS-5 cells with peripheral blood mononuclear cell (PBMC) samples obtained from B-CLL patients. Mixed cells and transwell co-cultures experimental settings were used to highlight and distinguish the possible dependence of the activation mechanism on cell contact. In both settings, the percentage of Annexin V/PI-positive (apoptotic) cells was considerably reduced in HS-5/PBMC co-culture of 7 newly diagnosed patients (Table 1) (Fig. 1C, D), thus confirming a stromal fibroblasts effect on tumor cell survival. In detail, it was observed a significant inhibition of spontaneous apoptosis, ranging from 51% to 80%, in co-cultured B-CLL cells growing in contact with untreated or NT- siRNA transfected HS-5 cells (Fig. 1C). This effect significantly decreases, or vanishes, in all B-CLL samples co-cultured with BAG3 silenced HS-5 cells, thus suggesting a functional role of BAG3 in tumor survival supported by stromal fibroblast. To further dissect the complex tumor survival mechanism and identify their BAG3-dependent elements, a series of patient-derived B-CLL cells were studied in transwell co-cultures, to discover the role of possible soluble mediators. In this experimental setting, the stromal HS-5 cells significantly lowered the spontaneous apoptosis of B-CLLs, with a reduction efficacy ranging from 50% to 92%. Again, as previously shown in mixed cell cultures, the silencing of BAG3 expression markedly reversed the pro-survival effect (Fig. 1D). These pieces of evidence suggested that BAG3 in fibroblasts serves its primary function as an important ancillary factor supporting cellular signaling mechanisms mediated by soluble molecules.

**Table 1:** Patients’ characteristics at baseline.

Characteristics	CLL, *N* = 7 (%)
Mean age, years old (range)	69 (53–80)
Sex (M/F)	5/2
Disease stage—Rai system	
0	4 (57)
1	2 (29)
2	–
3	1 (14)
4	–
Mean Hb, g/dl (range)	14.3 (12.5–16)
Mean WBC, cells/μl (range)	23,732 (13,500–50,300)
Mean ANC, cells/μl (range)	4446 (3000–7249)
Mean ALC, cells/μl (range)	11,095 (8220–16,700)
Mean PLT/μl (range)	147,250 (6000–218,000)
Mean LDH, U/l (range)	296 (147–654)
Median follow-up, months (range)	17.8 (3.7–51.3)
IGHV mutational status	
Unmutated	1 (14)
Mutated	2 (29)
Not performed	4 (57)
TP53 mutational status	
Wild type	4 (57)
Mutated	–
Not performed	3 (43)
Immunophenotyping	
CD38+/−	0 (0)/7 (100)
CD49d+/−	0 (0)/7 (100)
FMC7+/−	4 (57)/3 (43)
CD20+/−	0 (0)/7 (100)
CD11c+/−	2 (29)/5 (71)
CD200+/−	6 (86)/1 (14)
CD43+/−	5 (71)/2 (29)
SmIg−	4 (57)
κ/λ	2 (29)/1 (14)

*CLL* chronic lymphocytic leukemia, *Hb* hemoglobin, *WBC* white blood cells, *ANC* absolute neutrophil count, *ALC* absolute lymphocyte count, *PLT* platelets, *LDH* lactate dehydrogenase, *IGHV* immunoglobulin heavy chain gene, *SmIg* membrane surface immunoglobulin.

**Fig. 2:**
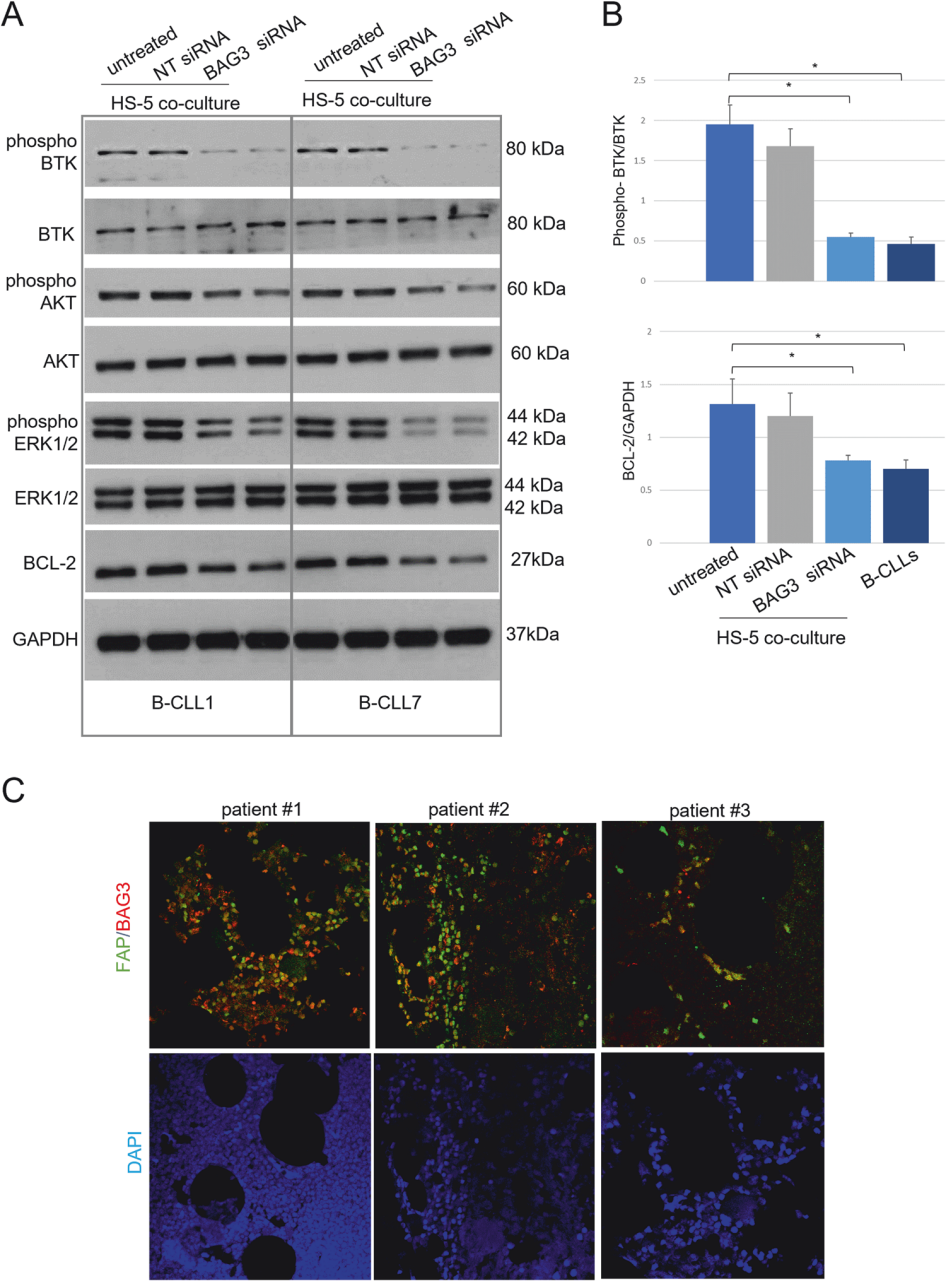
BAG3 protein is expressed in stromal FAP-positive cells in advanced-stage patients’ specimens and regulates in co-cultures survival signaling pathways in B-CLLs. **A** HS-5 cells were transfected with either BAG3 siRNA or NT siRNA. After 16 h patient-derived B-CLL cells were exposed to transfected HS-5 in transwell co-cultured or alone (B-CLL1 and B-CLL7). The B-CLL cells were collected after 120 h, and whole-cell extracts were analyzed by immunoblotting with the indicated antibodies. **B** Sample densitometry data are expressed as fractions of the phospho-BTK/BTK and BCL-2/GAPDH ratios. Student’s *t*-test was used to calculate *p* values, which are represented as *p < 0.05 > 0.01, **p < 0.01 > 0.001, and ***p < 0.001.1. **C** Representative images of BAG3 (red) and FAP marker (green) expression in infiltrated bone marrow specimens obtained by immunofluorescence. Yellow signals are referred to as merged images. The 63× magnification was used during image acquisition.

To additionally study the regulatory pattern of B chronic leukemia cell survival, it was hypothesized and verified the involvement of some key players of known signaling pathways in the still unclear pro-survival mechanism in which BAG3 seems to play a significant role. Among these, Bruton’s Tyrosine Kinase (BTK)-located downstream of the B cell antigen receptor (BCR)–orchestrates a complex series of cellular responses essential for leukemic cell survival [22]. The chronic activation of BTK in CLL cells determines the activation of AKT (Protein Kinase B) and ERK (extracellular signal-regulated kinase), which in turn influences cellular proliferation, differentiation, and survival. This constant and aberrant signal results in the dysregulated and malignant characteristic of BCL-2 (B-Cell Lymphoma 2), due to a significant anti-apoptotic activity dependent on its increased expression [23]. Figure 2A, B shows an immunoblotting analysis displaying the levels of the above-mentioned molecules in two samples of B-CLL cells cultured alone or in the presence of HS-5 cells for 120 h. The obtained results showed that the phosphorylation of BTK, AKT, and ERK, was higher in leukemic cells when co-cultured in transwell with HS-5 (Fig. 2A). Conversely, BAG3 silencing in HS-5 cells markedly reversed the enhancement of BTK phosphorylation and BCL-2 protein levels in B-CLL cells (Fig. 2B), which is also consistent with the apoptotic trends in the same experimental settings. The obtained experimental pieces of evidence show that BAG3 plays an important role in the HS-5-mediated reduction of apoptosis and demonstrates its involvement in the intrinsic survival pathways of B-CLL tumor cells supported by the essential interplay with the stromal microenvironment. To further validate our hypothesis, we evaluated BAG3 expression in infiltrated bone marrow specimens from B-CLL patients. We observed cells co-expressing BAG3 and FAP, a recognized marker of the nurturing fibroblastic niche [9, 24], that shows the presence in the specimens of BAG3-expressing fibroblasts in the advanced stage of the disease (Fig. 2C).

### BAG3-dependent modulation of cytokine network in stroma- B-CLL co-cultures

**Fig. 3:**
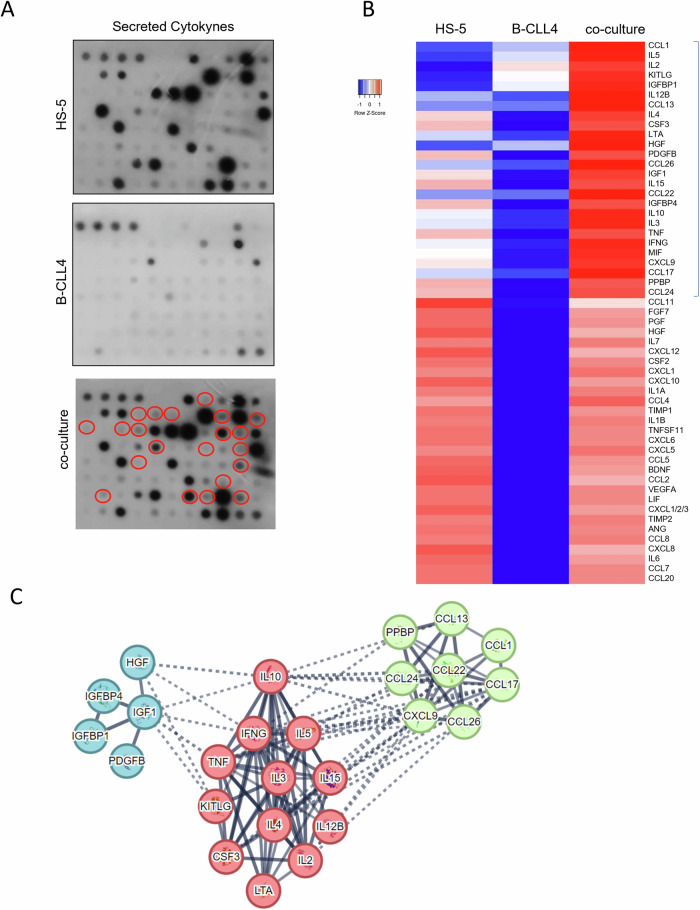
Cytokine network in stroma-B-CLL co-cultures. **A** Human Cytokine-specific Antibody Arrays 5 (RayBio C-Series) were incubated with 1 ml supernatants of HS-5, B-CLL4, and co-culture cells, following the manufacturer’s instructions. The panel shows images obtained by similar exposures. Red circles depict cytokines upregulated in co-cultures. **B** Heatmap representing different expressions of secreted cytokines in HS5, B-CLL4, and co-cultures. **C** STRING analysis (*k* = 3) of the cytokines upregulated in co-cultures.

To study the variations of soluble factors composition in the medium of stromal fibroblasts co-cultured with chronic B leukemia cells, the levels of different cytokines, crucially involved in cell signaling patterns, were analyzed in a comprehensive immunoblot panel. The cytokines contents were determined in the supernatants of three different cell culture settings: (1) HS-5 cells; (2) patient B-CLL4 cells; and (3) HS-5/B-CLL4: a co-culture in the transwell plate system. The intensities of the dots on the immunoblots were quantified by densitometric analysis of the digitized images (Fig. 3A). The results resumed as a heatmap in Fig. 3B showed the presence of two sets of homogeneously released cytokines. A first set includes several cytokines, undetectable in HS-5 and B-CLL4 monocultures and present only in the co-cultures media. This evidence suggests that the interaction of stromal fibroblasts and leukemia cells triggers the production of specific cytokines and specific cellular crosstalk in cell co-cultures. The second set includes cytokines that are stably expressed by stromal fibroblasts, both in monoculture and in co-cultures with leukemia cells. The data analyzed by using the software STRING available online, showed that all of the cytokines up-regulated in the co-cultures belong to three interconnected protein clusters: interleukins (IL-2, IL-3, IL-4, IL-5, IL-10, IL-12B, IL-15, KITLG, IFN-gamma, LTA, MIF, TNF-alpha, and CSF3), chemokines (CCL1, CCL13, CCL17, CCL22, CCL24, CCL26, CXCL9, and PPBP) and growth factors (HGF, IGF1, IGFBP1, IGFBP4, and PDGFB). As far as the STRING analysis is concerned, a strong association was found between members within each cluster, as represented in Fig. 3C. This evidence allows us to speculate on the possible biological meaning of the specific and coordinated signals produced in such a cellular environment. In fact, the cytokines up-regulated in co-cultures most probably enhance leukemic clone formation by regulating cellular proliferation and apoptosis, since specific cytokines, such as IL-2, IL-4, IL-10, IL-15, and TNF-alpha [25], can influence the survival of B-CLL cells in vivo. Furthermore, among up-regulated chemokines, CCL1, CCL17, and CCL22 can recruit T helper 1 (Th1) cells that support CLL activation and proliferation [26, 27]. Eotaxins (specifically CCL24 and CCL26) have also been correlated to cancer development through M2 macrophage polarization, angiogenesis, invasion and migration, and recruitment of eosinophils [28]. Finally, it has been shown that IGF [29, 30], HGF [31], and PDGFB [32], ligands of RTKs (Receptor Tyrosine-Kinase) play a role in CLL B-cell survival. About the set of cytokines secreted by both stromal fibroblasts and the co-culture, as expected, it was detected CXCL12 (SDF-1: Stromal Derived Factor-1), a chemokine that promotes cell survival by binding its specific receptor CXCR4, particularly present and highly expressed in CLL and other of blood malignancies. This signaling pathway not only improves cell survival, but it also influences the migration of leukemia cells into lymphoid tissue and bone marrow [33].

**Fig. 4:**
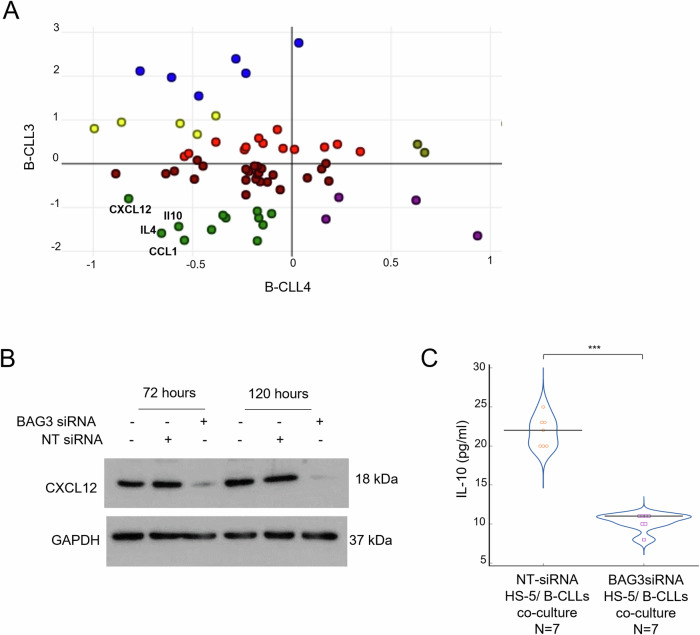
BAG3 silencing impacts cytokines expression profile. **A** Cluster visualization analysis (*k* = 6) in BAG3-silence HS-5 co-cultured with B-CLL3 and B-CLL4 leukemic patients’ samples. Cytokines co-expressed in the lower left quadrant represented soluble factors lowered by BAG3-silenced fibroblasts. **B** HS-5 cells were transfected using the methods described above. Cells were harvested at 72 h and 120 h, and total cell extracts were analyzed by western blotting with an anti-CXCL12 polyclonal antibody; an anti-GAPDH antibody served as a loading control. Student’s *t*-test was used to calculate *p* values, which are represented as *p < 0.05 > 0.01, **p < 0.01 > 0.001, and ***p < 0.001. **C** HS-5 cells were transfected with either BAG3 siRNA or NT siRNA. After 16 h, patient-derived B-CLL cells were exposed to transfected HS-5 in transwell co-culture conditions. After 120 h, IL-10 content in supernatants was measured using ELISA. Data are presented as mean ± S.D. of duplicate experiments.

**Fig. 5:**
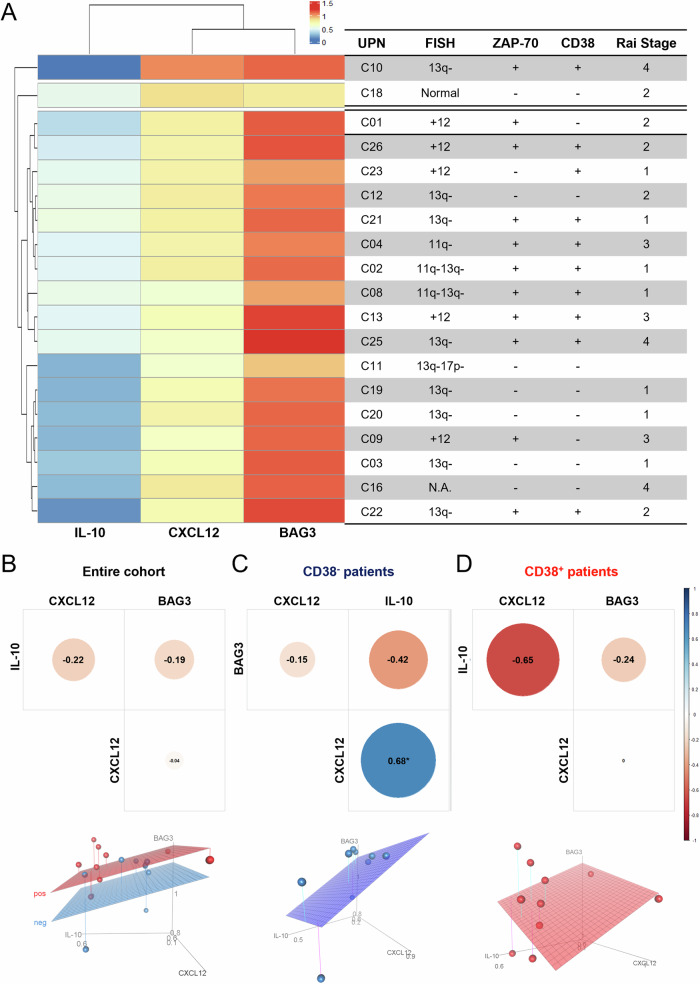
BAG3, CXCL12 and IL-10 gene expression correlations in a cohort of B-CLL patients. **A** Gene expression profiling of BAG3, CXCL12, and IL-10 from the GSE21029 dataset analysis of 19 B-CLL bone marrow specimens represented as a heatmap with related clinical, molecular, and phenotypical features. Pearson correlation analysis (upper graphs) and 3D scatter plot with regression surface (bottom graphs) of BAG3, CXCL12, and IL-10 in the entire cohort **B**, with two regression surfaces based on CD38 negativity [blue plan] or positivity [red plan]), in CD38 negative (**C**) and in CD38 positive (**D**) patients, *p < 0.05.

To better understand the impact of stromal fibroblasts BAG3 silencing on the intercellular signaling of stromal and neoplastic cells grown in mixed cultures, cytokine concentrations in co-culture media were compared to their concentrations in control cultures of two distinct samples of patient-derived cells (B-CLL3 and B-CLL4). A cluster analysis of the obtained data resulted in an in-depth description of the cytokine expression profile changes caused by BAG3 silencing (Fig. 4A). In the two patient-derived B-CLL samples the analysis showed that the levels of some released chemokines and cytokines decreased when co-cultured with BAG3 silenced stromal fibroblasts. Among them CXCL12 showed lower levels as well as IL-10, IL-4, and CCL1 that were up-regulated in co-cultures with non-silenced stromal fibroblast (Fig. 4A). As previously mentioned, CXCL12 is a major driver of stromal fibroblast supportive activity in B-CLL and the observed down-modulation in BAG3 silenced co-cultures prompted us to further study the involvement of BAG3 in its intracellular regulation. Immunoblot experiments in BAG3-silenced cells showed a similar decrease in HS-5 cells of BAG3 and CXCL12 protein levels at the two tested time points (Fig. 4B). It is worth noting that CXCL12/CXCR4 signaling pathway in the CLL microenvironment leads to IL-10 synthesis, as demonstrated by its increased levels found in sera of B-CLL patients [34]. The stimulation of the CXCR4-STAT3 pathway by CXCL12 expressing fibroblasts induces Il-10, which is involved either in immunosuppression in patients [35] or autocrine survival signaling in neoplastic cells [36]. To evaluate the effects of the CXCL12 decrease, the changes in IL-10 level in the supernatant of all B-CLL patients’ co-cultures were measured by ELISA assay. The results showed a strong and significant decrease of IL-10 in all B-CLL cells co-cultured with BAG3-silenced HS-5 (IL-10 mean ± S.D.: 10.5 ± 1.3) when compared to controls (IL-10 mean ± S.D.: 21.9 ± 1.8), thus proving, or letting infer, a sound correlation between BAG3 expression in stromal fibroblasts and IL-10 levels in the TME (Fig. 4C). This correlation was further confirmed by the analysis of a published bone marrow gene expression data available for 19 B-CLL untreated patients (GSE21029). Using this dataset, a linear regression analysis predicted a positive linear correlation between CXCL12 and BAG3 (*R* = 0.524, *p* < 0.001), as well as for CXCL12 and IL-10 (*R* = 0.707, *p* < 0.001) and for BAG3 and IL-10 (*R* = 0.385, *p* < 0.05). Gene expression, tumor molecular features (FISH results, ZAP-70 and CD38 positivity), and RAI stage for each patient are reported in Fig. 5A. Next, Pearson correlation analysis between each gene was performed on the entire B-CLL cohort confirming a tendency of correlated expression between studied genes (Fig. 5B). Interestingly, when patients were divided based on CD38 positivity (>30% of expression by flow cytometry), an opposite gene expression profile was observed. Indeed, CD38 negative patients showed a significantly direct correlation between CXCL12 and IL-10 expression (*R* = 0.68; *p* = 0.0434) (Fig. 5C), while CD38 positive patients displayed a strong inverse correlation (*R* = −0.65; *p* = 0.0427) (Fig. 5D). These results confirmed our findings, as all patients included in our cohort were negative for CD38 antigen and showed a positive correlation between CXCL12 and IL-10 expression. Adhesion molecules are important in CLL pathogenesis as they allow direct interaction between leukemic cells and TME, or indirectly induce cytokine release, including CXCL12, that promotes tumor growth, migration, homing, and survival [37, 38]. High expression levels of ZAP-70 and CD38 are associated with poor outcomes in CLL patients, and CD38 positivity is frequently related to more severe clinical manifestations [39], and to simultaneous CD49d and CD11c (other two adhesion molecules) positivity. CD38-positive CLL patients are more likely to receive chemotherapy with a shorter time to treatment and progression-free survival compared to CD38-negative subjects. Others have demonstrated a strong association between CD38 expression and CXCL12 signaling activation, likely because of physical proximity on the cell surface, and CD38 binding with agonistic monoclonal antibodies can induce CXCL12 pathway, while its blocking abolishes this chemokine effects and CLL homing to lymphoid organs in a mouse model [40]. Therefore, our results from in vitro studies and findings from published datasets added evidence to the strong interaction between CD38 and CXCL12, together with IL-10 and BAG3, and could be related to a worse prognosis [41]. Then, the study of clinical specimens revealed a statistically significant correlation between BAG3 levels and the chemokine CXCL-12/ cytokine IL-10 signaling pathway. This evidence supports a coordinated mechanistic relationship between survival molecular pathways ending in the proliferation and sustenance of neoplastic cells in human tissues and highlights the importance of BAG3 as a key driver in the pathophysiology and cell biology of B-CLL disease.

## Discussion

CLL patients who are resistant to the covalent inhibitors BTK and BCL2 present a new clinical challenge. In fact, current medications showed a limited and transient therapeutic efficacy which results in a reduced overall survival and makes these patients ideal candidates for clinical studies. It is indeed necessary to develop new treatment strategies capable of interfering with different mechanisms of cell survival and proliferation, particularly those able to interfere with new molecular targets. The complex interplay between B-CLL cells and the heterogeneous stromal milieu emphasizes the importance of determining the molecular processes controlling this relationship. The broad heterogeneity of CAFs most probably stems from their diverse origins. This complexity is further compounded by the fact that no single marker is uniquely expressed by CAFs, making it difficult to identify pure CAF populations, and their functional pro-tumoral or tumor-suppressing role in bone marrow. Indeed, the expression of BAG3 protein in CAF subtypes within the leukemic stroma is worthy of further investigation and could have significant implications for the development of tailored treatment methods aiming at interrupting the supporting network that drives leukemia progression [9, 24]. In this context, fibroblasts produce CXCL12, a chemokine required for tissue homing. This chemotactic gradient attracts CLL cells, which exhibit high amounts of the chemokine receptor CXCR4. Aside from its chemoattractant activity, CXCL12 has modest pro-survival effects and plays an important role in the stroma-mediated protection of CLL cells from apoptosis, both spontaneous and chemotherapy-induced [42]. Ulocuplumab, a completely human IgG4 anti-CXCR4 antibody, able to inhibit CXCL12 binding to its receptor, has shown promise in preclinical research and early-stage clinical trials for various hematological malignancies [43]. It has been shown to block the migration of CLL cells when CXCR4 is activated, induce apoptosis, and decrease the development and migration of cancer cells. Despite these promising findings, Ulocuplumab is still not widely used in CLL or other kinds of chronic leukemia. This could be due to the efficacy of Ulocuplumab which may rely on CXCR4 mutations in leukemia cells, to the adverse effects such as reversible thrombocytopenia, which must be closely monitored, and to the fact that new medications in chronic leukemia must exhibit considerable improvements over existing therapy to gain widespread use [43]. Here, a clear link has been described, between the expression of BAG3 and CXCL12 in B-CLL patient-derived in vitro co-cultures, as well as in patient-derived bone marrow tissues. Furthermore, in the stromal fibroblasts, the BAG3 targeting strategy effectively lowered tumor cell survival by modulating CXCL12 levels while nurturing an unfavorable cytokine environment. Finally, the BAG3 protein, with its diverse regulatory activities in major intracellular processes, as well as its roles as a mediator of intercellular signaling [12], could be a target of significant interest in hematological disorders.

## Materials/subjects and methods

### Patient sample collection

Whole peripheral blood (PB) specimens were collected in ethylenediaminetetraacetic acid (EDTA) tubes for immunophenotyping and in vitro studies from a total of seven subjects after informed consent obtained in accordance with the Declaration of Helsinki and protocols approved by our local Ethics Committee “Campania Sud” (Brusciano, Naples, Italy; prot./SCCE n. 24988). Patients received a diagnosis of CLL according to current guidelines [PMID: 29540348] at the Hematology and Transplant Center, University Hospital “San Giovanni di Dio e Ruggi d’Aragona” of Salerno, Italy. Clinical characteristics at enrollment are summarized in Table 1. All subjects were on wait-and-watch monitoring. Diagnostic immunophenotyping was performed on PB specimens by flow cytometry using the following antibodies according to manufacturers’ instructions: CD45; CD4; CD8; CD3; CD56; CD19; CD5; CD23; CD10; CD11c; CD20; CD103; CD38; CD43; CD49d; CD200; FMC7; SmIg-Kappa; and SmIg (all from Beckman Coulter). Sample acquisition was carried out on a 10-color three-laser Beckman Coulter NaviosEX Flow Cytometer (Beckman Coulter), and post-acquisition analysis was performed using Kaluza Analysis Flow Cytometry software v2.1.1 (Beckman Coulter). For immunofluorescence evaluation, bone marrow (BM) biopsies from three B-CLL patients with tumor infiltration were included.

### Cell cultures and cell isolation

Human bone marrow stromal (HS-5) cell lines were purchased from ATCC (CRL-11882) and were cultured in Dulbecco’s modified Eagle’s medium (DMEM) with 10% FBS 2 mmol/l L-glutamine, 100 U/ml penicillin, and 100 microg/ml streptomycin (Sigma-Aldrich). PBMCs from B-CLL patients were isolated by density-gradient centrifugation over Ficoll-Paque (GE Healthcare) and centrifuged at 400 g for 30 min. The isolated mononuclear cells (PBMCs) were washed three times with PBS 1×. After counting, the cells were utilized immediately or grown in full RPMI 1640 with 10% heat-inactivated fetal bovine serum (FBS), 2 mmol/l l-glutamine, 100 U/ml penicillin, and 100 microg/ml streptomycin (Sigma-Aldrich). All tests were carried out using freshly obtained B-CLL cells. The purity of the samples was determined by flow cytometry and ranging of CD5^+^CD19^+^ cells from 60% to 90%. In co-culture experiments, primary B-CLL cells were grown in RPMI-1640/10% FBS in 24- or 6-well plates at a density of 1 × 10^6^ cells/ml (in contact or transwell co-culture) over a monolayer of HS-5 cells (80% confluence) treated with BAG3 siRNA or NT siRNA. B-CLL and stromal cells were seeded in a 20:1 ratio. Trypan blue exclusion was used to determine cell viability, followed by counting in a Burker chamber. All cultures were incubated at 37 °C under a humidified 5% CO_2_ environment.

### Transfections

HS-5 cells were transfected with either a siRNA targeting bag3 mRNA (5’-AAGGUUCAGACCAUCUUGGAA-3’) or an NT siRNA (5’-CAGUCGCGUUUGCGACUGG-3’). For transfection, cells were plated in 24-well plates (5 × 10^4^/well) or 6-well plates (2 × 10^5^/well) to achieve 80% confluency and incubated at 37 °C in 5% CO_2_. Cells were transfected with a final siRNA dose of 25 nM using TransIT-X2 reagent (Mirus Bio LLC- #MIR 6010) for 72 h and 120 h, according to the manufacturer’s instructions. Western blot analysis was used to determine transfection efficiency in each experiment.

### Western blotting and antibodies

Protein concentrations were determined using a lysis buffer (20 mM HEPES pH 7, 150 mM NaCl, 1 mM EGTA, 1% IGEPAL) including a protease inhibitor cocktail (1 mM phenylmethylsulfonyl fluoride, 1 mg/ml pepstatin A, 2 mg/ml aprotinin). The protein level was measured using the Bradford assay. Thirty micrograms of total protein were electrophoretically transferred to a nitrocellulose membrane after running it on 12% SDS-PAGE gel. Nitrocellulose blots were blocked with 10% nonfat dry milk in TBST buffer (20 mM Tris-HCl pH 7.4, 500 mM NaCl, and 0.1% Tween 20) before being incubated with the primary antibody in TBST containing 5% nonfat dry milk overnight at 4 °C. Immunoreactivity was identified by incubating with horseradish peroxidase-conjugated secondary antibody and ECL reagents in sequence. Densitometry of bands was carried out using ImageJ software (NIH, USA). The area under the curves, relative to a band, was calculated, and the background was subtracted from the results. Santa Cruz Biotechnology Inc. (Santa Cruz, California, USA) provided antibodies that recognize ERK1/2 (K-23: sc-94) and GAPDH (sc-25778). Abcam (Cambridge, UK) provided antibodies for α-SMA (ab7817), phospho-BTK (pY551-ab52192), and CXCL12 (ab9797). Proteintech (Manchester, UK) provided antibodies that recognize BTK (#21581) and BCL-2 (#68103-1-Ig). Antibodies to phospho-ERK 1/2 (Thr202/Tyr204: #9101), AKT (#9272), and phospho-AKT (ser 473: #9271) were bought from Cell Signaling Technology, Inc. (Danvers, MA). A polyclonal anti-BAG3 antibody produced in our lab was used to detect BAG3 levels.

### Apoptosis detection

Primary B-CLL cells were placed in 24-well plates at 1 × 10^6^ cells/ml alone or in co-culture with HS-5 cells. After 120 h, primary B-CLL cells were collected and treated with APC-anti-CD19 (BioLegend; #392504) for 15 min, then with FITC-annexin-V and propidium iodide (PI) for 15 min in the dark at room temperature. APC- conjugated IgG isotypic negative control (BioLegend, #400121) was used to determine baseline fluorescence. The manufacturer’s instructions were followed while performing the assay with the FITC Annexin V Apoptosis Detection Kit with PI (BioLegend, #640914). Apoptotic cells were identified by flow cytometry (FacsVerse, Becton Dickinson). The fluorescence distribution was determined using a dot plot analysis (electronic adjustment was required to avoid overlapping of the two emission spectra), and the proportion of fluorescent cells in each quadrant was calculated. The percentages of live (annexin^−^/PI^−^), early apoptotic (annexin^+^/PI^−^), late apoptotic (annexin^+^/PI^+^), and necrotic (annexin^−^/PI^+^) cells were evaluated using FacsVerse from Becton Dickinson.

### Cytokine array

After 120 h, the conditioned medium from HS-5 and B-CLL monocultures or co-cultures were collected and centrifuged for 20 min at 2000 g at 4 °C; supernatants were then transferred and centrifuged for 30 min at 10,000 g at 4 °C to remove cellular debris. Cytokine levels were measured in cleared supernatants using the RayBio C-Series Human Cytokine Antibody Array C5 (RayBiotech, Norcross, GA; # AAH-CYT-5), according to the manufacturer’s instructions. Membranes were placed in a tray with a blocking buffer and incubated for 30 min at room temperature (RT). After blocking, 1 ml of conditional medium was added to the membranes and incubated at 4 °C overnight. The membranes were washed twice with buffer, then incubated with Biotinylated Antibody Cocktail overnight at 4 °C. After washing, the membranes were incubated with HRP-Streptavidin Concentrate for 2 h at room temperature, rinsed twice, and placed in detection buffer for 2 min. Signals were detected using the ChemiDoc MP Imaging System (BIO-RAD). At least two exposures for each blot were analyzed by ImageJ (NIH, Bethesda, MD), and relative cytokine levels were determined by densitometry. The quantitative data received from the cytokine array analysis were converted to a heatmap utilizing the web tool available at http://heatmapper.ca. The rows of the heatmap represent individual cytokines, the columns reflect different situations, and the colors represent cytokine expression levels, with a color gradient (from blue to red) indicating low to high expression. The IL-10-specific content in the supernatant was quantified using an ELISA kit according to the manufacturer’s instructions (Thermo Fisher).

### Protein interaction network analysis

The Search Tool for the Retrieval of Interacting Genes/Proteins (STRING version 9.0, http://string90.embl.de/) was used to investigate interactions between cytokines with varied expression levels in the HS-5/B-CLL4 co-culture. STRING analysis was performed with good confidence (score 0.7). Cluster analysis of up-regulated cytokines was performed using k-means with a value of *k* = 3. A cluster analysis was also applied to cytokine changes in BAG3-silenced fibroblast co-cultures in two different patients. Cytokine levels were compared using a free online web tool available at https://www.statskingdom.com/about.html.

### Immunofluorescence

For paraffin-embedded sections, immunofluorescence protocol included deparaffinization in Clear-Rite™ 3 (ThermoScientific, Waltham, MA, USA), rehydration through descending degrees of alcohol up to water, non-enzymatic antigen retrieval in sodium citrate buffer 10 mM, 0.05% Tween, pH 6.0, for 40 min in a pressure cooker at 95 °C. After washing, non-specific binding was blocked with 10% normal goat serum (NGS) in PBS 1× 1 h, RT. Sections were then incubated with an anti-FAP (Abcam, ab28244—3 μg/ml) and a murine anti-BAG3 mAb (3 μg/ml) developed in our lab, overnight at 4 °C in a humidified chamber. After another washing step, the sections were incubated with the secondary antibodies (DyLIGHT 488-conjugated AffinePure Goat anti-rabbit and DyLIGHT 649-conjugated AffinePure Goat anti-mouse from Jackson ImmunoResearch, Cambridge, UK, used at 1:200 dilution). Nuclei were counterstained with 1 μg/ml DAPI (Molecular Probes, Eugene, Oregon, USA). The slides were then coverslipped using an aqueous mounting medium and analyzed using a confocal laser scanning microscope (Leica SP5, Leica Microsystems, Wetzlar, Germany). The images were captured in sequential scan mode with consistent acquisition settings (laser intensities, gain photomultipliers, pinhole aperture, ×63 objective) for both experimental and control samples. To prepare the figures, brightness, and contrast were adjusted to maintain a light cellular fluorescence background, allowing for the visualization of the lowest fluorescence intensity features.

### Statistical analysis

Results are presented as means ± S.D. The data were analyzed using the Student’s *t*-test in Graph-Pad Prism statistical software version 4.01 (La Jolla, CA, USA) and graphs obtained by Excel or MedCalc Software. Heatmaps, correlograms, and 3D scatter plots were made with R software (Rstudio v.2022.07.1) using *corrplot*, *ggplot2*, *rgl*, and *car* packages. *p* Values ranging from 0.01 to 0.05, 0.001 to 0.01, or 0.001 were classified as significant (*), very significant (**), or extremely significant (***), respectively.

### Supplementary information

western blot original

## Data Availability

The data that support the findings of this study are available from the corresponding author, [AR], upon reasonable request.
