# Supplementary figures and images for: Tuning the B-CLL microenvironment: evidence for BAG3 protein- mediated regulation of stromal fibroblasts activity
Source: Cell Death Discov. 2024 Aug 28;10:383. doi: 10.1038/s41420-024-02153-6 (PMC11358476; doi:10.1038/s41420-024-02153-6)

Figure 1A

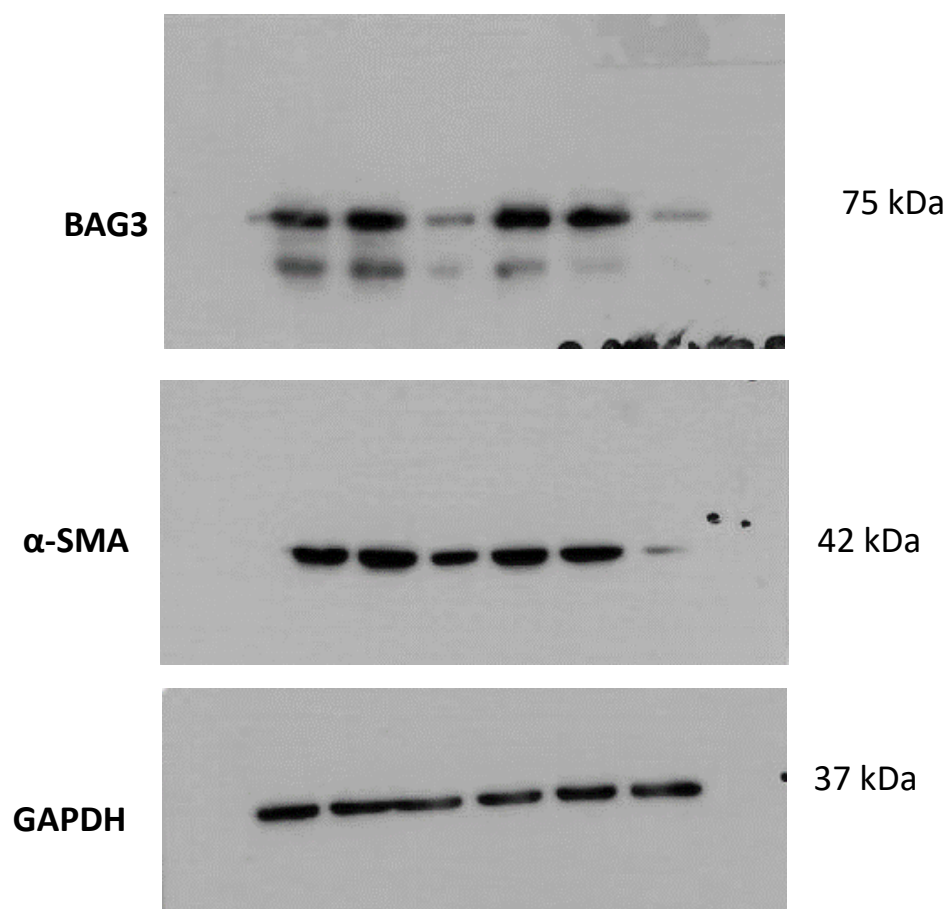

Figure 2A

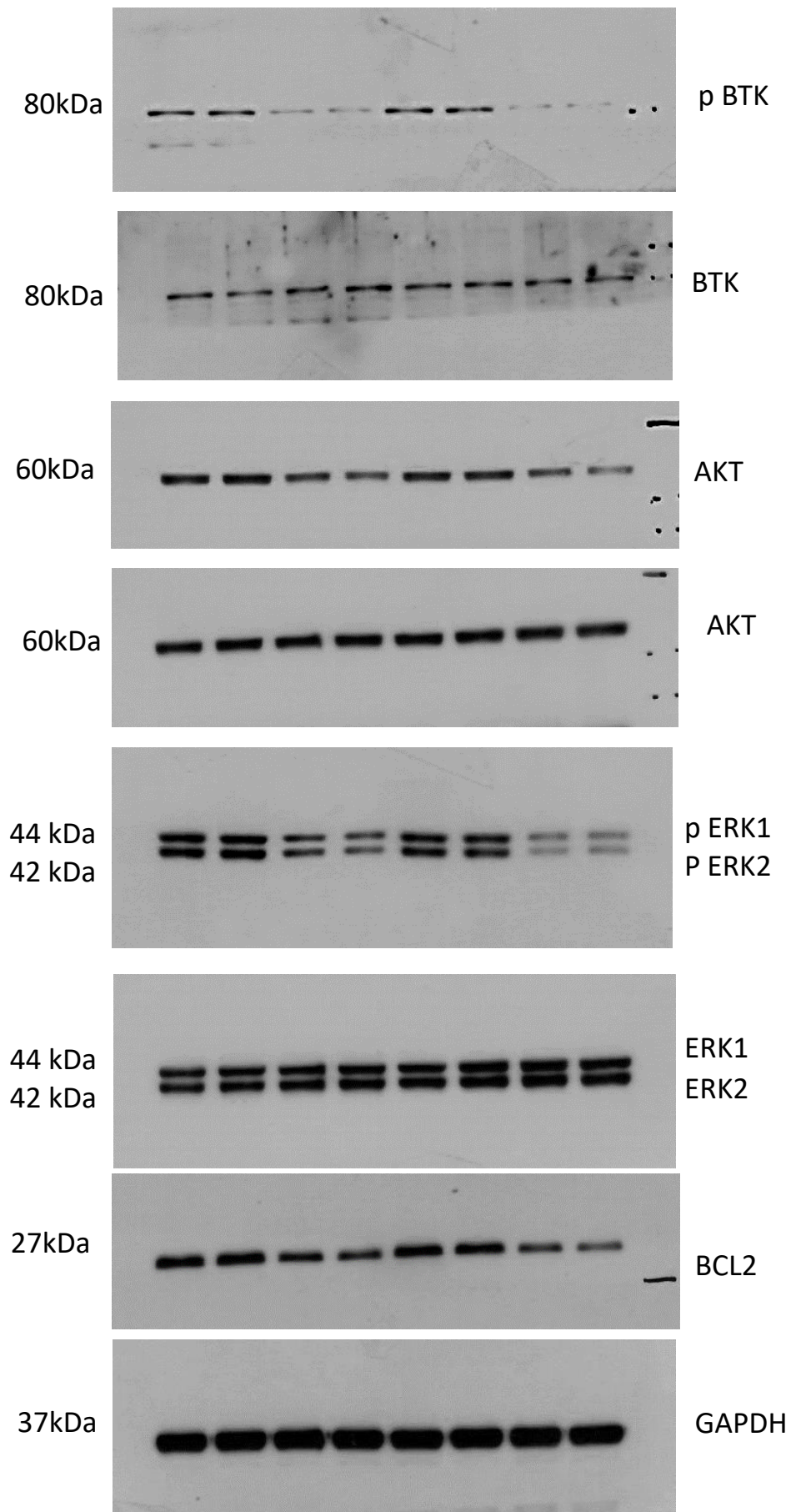

Figure 4C

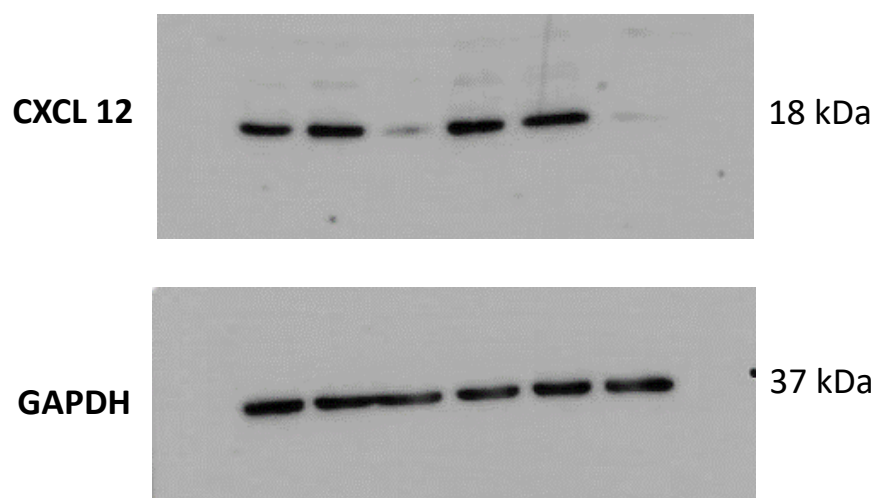

Supplement: Supplementary file 1 — western blot original [file 41420_2024_2153_MOESM1_ESM.pdf]
